# Missed opportunities for HIV testing in patients newly diagnosed with HIV in Morocco

**DOI:** 10.1186/s12879-020-05711-2

**Published:** 2021-01-11

**Authors:** Latifa Marih, Victoire Sawras, Juliette Pavie, Mustapha Sodqi, Mourad Malmoussi, Noura Tassi, Rajaa Bensghir, Samira Nani, Ahd Oulad Lahsen, Didier Laureillard, Kamal Marhoum El Filali, Karen Champenois, Laurence Weiss

**Affiliations:** 1 Service des maladies infectieuses, Centre Hospitalier Universitaire Ibn Rochd, Casablanca, Morocco; 2 Inserm, IAME, UMR 1137, Paris, France; 3 Université Paris Nord, Sorbonne Paris Cité, Paris, France; 4 Hôpital Bichat Claude Bernard, AP-HP, Paris, France; 5 Service d’Immunologie Clinique, Hôpital Européen Georges Pompidou, AP-HP, INSERM UMR 1149, 20, rue Leblanc, 75015 Paris, France; 6 Service des maladies infectieuses, Hôpital Hassan II, Agadir, Morocco; 7 Service des maladies infectieuses, Centre Hospitalier Universitaire Mohamed VI, Marrakech, Morocco; 8 Laboratoire d’épidémiologie, Faculté de Médecine et de Pharmacie, Casablanca, Morocco; 9 Service de Maladies Infectieuses, CHU de Nîmes, Nîmes, France; 10 Université Paris Descartes, Sorbonne Paris Cité, INSERM U976, 20, rue Leblanc, 75015 Paris, France

**Keywords:** HIV testing, Missed opportunities, HIV indicator conditions, Late HIV diagnosis, Key populations

## Abstract

**Background:**

In Morocco, of the estimated 29,000 people living with HIV in 2011, only 20% were aware of their HIV status. More than half of diagnoses were at the AIDS stage. We assumed that people who were unaware of their infection had contacts with the healthcare system for HIV indicators that might prompt the healthcare provider to offer a test. The aim was to assess missed opportunities for HIV testing in patients newly diagnosed with HIV who accessed care in Morocco.

**Methods:**

A cross-sectional study was conducted in 2012–2013 in six Moroccan HIV centers. Participants were aged ≥18, and had sought care within 6 months after their HIV diagnosis. A standardized questionnaire administered during a face-to-face interview collected the patient’s characteristics at HIV diagnosis, HIV testing and medical history. Contacts with care and the occurrence of clinical conditions were assessed during the 3 years prior to HIV diagnosis. Over this period, we assessed whether healthcare providers had offered HIV testing to patients with HIV-related clinical or behavioral conditions.

**Results:**

We enrolled 650 newly HIV-diagnosed patients (median age: 35, women: 55%, heterosexuals: 81%, diagnosed with AIDS or CD4 < 200 cells/mm^3^: 63%). During the 3 years prior to the HIV diagnosis, 71% (*n* = 463) of participants had ≥1 contact with the healthcare system. Of 323 people with HIV-related clinical conditions, 22% did not seek care for them and 9% sought care and were offered an HIV test by a healthcare provider. The remaining 69% were not offered a test and were considered as missed opportunities for HIV testing. Of men who have sex with men, 83% did not address their sexual behavior with their healthcare provider, 11% were not offered HIV testing, while 6% were offered HIV testing after reporting their sexual behavior to their provider.

**Conclusions:**

Among people who actually sought care during the period of probable infection, many opportunities for HIV testing, based on at-risk behaviors or clinical signs, were missed. This highlights the need to improve the recognition of HIV clinical indicators by physicians, further expand community-based HIV testing by lay providers, and implement self-testing to increase accessibility and privacy.

**Supplementary Information:**

The online version contains supplementary material available at 10.1186/s12879-020-05711-2.

## Background

Late diagnosis of HIV infection, and therefore late access to antiretroviral treatment, leads to significant HIV-related morbidity and mortality, as well as an increase in onward HIV transmissions [1, 2]. In Morocco in 2011, of the estimated 29,000 people living with HIV, only 20% were aware of their HIV status. More than half of diagnoses were at the AIDS stage [3].

However, at this time, national guidelines recommended HIV testing for people who seek care with clinical signs suggestive of an underlying HIV infection and/or for people from key populations [4]. In 2011, although there was a low HIV prevalence in the general population (0.1%), people who inject drugs (PWIDs), men who have sex with men (MSM) and female sex workers (FSWs) were disproportionally affected by HIV with a prevalence of 11.4, 5.1 and 1.9%, respectively [5]. Considered together, these data highlighted insufficient access to HIV testing and failure of HIV strategies to reach those most at risk of HIV acquisition.

In other countries like France and Ivory Coast [6–9]**,** a large proportion of people who were unaware of their HIV infection had contacts with the healthcare system during the period of probable HIV infection. During these contacts, an HIV test was rarely offered by the healthcare provider even if the patients reported HIV indicators. Several diseases or clinical symptoms have been highlighted as HIV indicators since the undiagnosed HIV prevalence among the patients who suffer from them was > 0.1%, thus justifying routine HIV testing [10–12]. Identifying these missed opportunities to offer a test and quantifying the missed opportunities could guide policy makers in extending HIV testing guidelines. The aim of this study was to assess the missed opportunities for HIV testing in patients newly diagnosed with HIV who accessed care in Morocco.

## Methods

### Study design and population

A cross-sectional study was conducted between May 2012 and December 2013 among patients newly diagnosed with HIV in six Moroccan infectious diseases departments in Agadir (1), Casablanca (1), Marrakesh (2), Nador (1) and Oujda (1) (Fig. S1). These six centers accounted for more than 85% of the people in care for HIV in Morocco.

Each new patient was screened for eligibility at the first visit with the physician. Patients aged ≥18 who sought care in one of the participating centers within 6 months after HIV diagnosis and who signed the written informed consent were enrolled in the study. Patients diagnosed with HIV in a foreign country or living in Morocco for less than one year were excluded, given that the opportunities for testing were not in Morocco.

The study was approved by the Ethics Committee of Biomedical Research of the Faculty of Medicine and Pharmacy of Casablanca.

### Data collection

A standardized paper questionnaire [6] written in French was administered in French (or local languages when needed) by physicians within the 6 months following HIV diagnosis. Patients’ sociodemographic characteristics, HIV acquisition risk factors, and clinical and immunological data at HIV diagnosis were collected. Patients were asked retrospectively about their HIV testing history and contacts with the healthcare system during the 3 years prior to HIV diagnosis. For the purpose of this study, a contact with the healthcare system was defined as any contact with a general practitioner or other specialized clinician, hospital, emergency service, pharmacy, or any other care facility. A list of these care facilities was shown to patients to help them understand and remember their health trajectory.

The questionnaire was designed with a stopping criterion. Only patients who sought care during the 3 years prior to HIV diagnosis were asked about the occurrence of behavioral and clinical indicators. Patients reporting a behavioral indicator were asked if they had mentioned it to a healthcare provider when they sought care, whatever the reason. Patients with clinical indicators were asked if they sought care because of these indicators. In both cases, they were asked if the healthcare provider had offered them HIV testing at this visit.

### Outcome definitions

We defined a missed opportunity for HIV testing as a contact with the healthcare system within the 3 years prior to HIV diagnosis, during which the patient presented HIV-related clinical conditions or mentioned belonging to a high-risk group for HIV acquisition, and where the healthcare provider did not offer an HIV test.

Behavioral indicators included [5, 13, 14] were MSM, people having multiple sex partners or condomless sex with casual partners. (Too few PWIDs were included in the study to carry out an analysis in this specific subgroup.)

Clinical indicators were clinical conditions and diseases frequently associated with the chronic stage of HIV infection [4, 5, 9, 11]: generalized lymphadenopathy, seborrheic dermatitis, prurigo, onychomycosis, oral herpes, oral hairy leukoplakia, oral candidiasis, varicella zoster, unexplained weight loss ≥10%, recurrent or persistent diarrhea lasting for ≥1 month, unexplained fever for ≥1 month, recurrent bacterial infections, community-acquired pneumonia, pulmonary tuberculosis, viral hepatitis B or C and sexually transmitted infections. As we studied missed opportunities for HIV testing, and not missed opportunities for HIV diagnosis, AIDS-defining opportunistic infections, with the exception of pulmonary tuberculosis, were excluded. HIV-related conditions occurring < 4 months prior to HIV diagnosis were considered to be directly related to diagnosis. Therefore, testing opportunities were assessed in patients with HIV-related conditions occurring between 3 years and 4 months prior to HIV diagnosis.

### Statistical analysis

Descriptive statistical methods were used to describe the study population and HIV testing opportunities. Qualitative variables were compared using the Chi-square test or Fisher’s exact test when appropriate and quantitative variables were compared using the Wilcoxon rank-sum test; *p*-values ≤0.05 (two-tailed) were considered significant. Participants’ characteristics and the HIV testing opportunities were compared by sex. Among patients who sought care for their HIV clinical indicators, those with a missed opportunity were compared to those who were offered a test. In the same way, patients who sought care for HIV-related clinical indicators were compared to those who did not.

Statistical analyses were performed using SAS 9.4 software (SAS Institute Inc., Cary, NC, USA).

## Results

### Participants’ characteristics

Six-hundred and fifty newly HIV-diagnosed patients were included in the study, 356 (55%) of whom were women. Their characteristics are detailed in Table 1. Compared to men, women were younger (34 versus 37, *p* = 0.0002), more likely to have no diploma (46% versus 20%, *p* <  0.0001), no professional activity (67% versus 20%, p <  0.0001) and no health insurance (82% versus 74%, *p* = 0.0001).

**Table 1:** Characteristics of patients newly diagnosed with HIV enrolled in the study (*n* = 650)

	Total (***n*** = 650)	Men (***n*** = 294)	Women (***n*** = 356)	***p***-value
**Age,** median (IQR)	35 (29–42)	37 (30–44)	34 (29–41)	0.0002
**Nationality,** Morocco^a^	638 (98%)	286 (97%)	352 (99%)	0.15
**Place of residence,** urban	531 (82%)	239 (81%)	292 (82%)	0.84
**Educational level**				< 0.0001
University	45 (7%)	34 (12%)	11 (3%)	
≤ High school	384 (59%)	201 (68%)	183 (51%)	
No diploma	221 (34%)	59 (20%)	162 (46%)	
**Professional status**				< 0.0001
Employed	335 (51%)	216 (74%)	119 (33%)	
Unemployed	297 (46%)	60 (20%)	237 (67%)	
Student	11 (2%)	11 (4%)	0	
Retired	7 (1%)	7 (2%)	0	
**Marital status**				< 0.0001
Married	286 (44%)	144 (49%)	142 (40%)	
Single	203 (31%)	128 (44%)	75 (21%)	
Divorced	116 (18%)	17 (6%)	99 (28%)	
Widowed	45 (7%)	5 (1%)	40 (11%)	
**Number of children,** median (IQR)	1 (0–1)	0 (0–1)	1 (0–1)	0.001
**Health insurance**				0.001
Public insurance^b^	94 (15%)	44 (15%)	50 (14%)	
Private insurance	47 (7%)	33 (11%)	14 (4%)	
Without insurance	509 (78%)	217 (74%)	292 (82%)	
**Sexual practices over the 3 years prior to HIV diagnosis**				< 0.0001
MSM	51 (8%)	51 (17%)	–	
Heterosexual	526 (81%)	223 (76%)	303 (85%)	
No sexual intercourse	65 (10%)	16 (5%)	49 (14%)	
Did not wish to answer	8 (1%)	4 (1%)	4 (1%)	
**Number of sexual partners**^**c**^**,** median (IQR)	1 (1–11)	4^d^ (1–24)	1 (1–4)	< 0.0001
**PWIDs**^**e**^	5 (0.8%)	4 (1%)	1 (0.3%)	0.18

*IQR* interquartile range, *MSM* men who have sex with men, *PWIDs* people who inject drugs

^a^ Foreigners (12): Algeria (1), Cameroon (1), Ivory Coast (2), Egypt (1), France (5), Guinea (1), Senegal (1)

^b^ Public insurance including CNOPS (national provident organizations fund, *n* = 24) and RAMED (medical insurance for economically disadvantaged people, *n* = 70)

^c^ 30 missing values

^d^ Median number of sexual partners among heterosexual and homosexual men

^e^ 1 missing value

Overall, considering sexual behavior, 81% of those included had heterosexual practices, 8% were MSM, and 10% reported no sexual intercourse within the 3 years prior to HIV diagnosis. The median number of sexual partners was 3 in heterosexual men, which was higher than in women (1, *p* <  0.0001) and lower than in MSM (14, Interquartile range (IQR): 3–50). A group of women, accounting for 11% of our study population, reported a higher number of sexual partners in the last three years (40, IQR: 20–100).

### Characteristics of HIV infection at diagnosis

At the time of diagnosis, 233 (36%) patients were at the AIDS stage as defined by the World health organization clinical staging classification [15] and 351 (54%) had a CD4 count < 200 cells/mm^3^ (median CD4/mm^3^: 169, IQR:48–348). No patients were diagnosed at the acute stage. Compared to women, men were more likely to be diagnosed with an AIDS-defining event (45% versus 29%, *p* <  0.0001) and a lower CD4 count (median CD4/mm^3^: 131 versus 200, p <  0.0001).

The main reasons for getting the test that revealed the HIV-positive status were: clinical signs (*n* = 382, 59%), voluntary testing (*n* = 133, 20%), and detection of a partner’s HIV-positive status (*n* = 79, 12%). As expected, the median CD4 count at diagnosis was higher in patients who discovered their HIV status through voluntary testing rather than those who experienced clinical signs (351 versus 77 cells/mm^3^, *p* <  0.0001). Of the 382 patients with clinical signs, the most common signs were unexplained weight loss ≥10% (43%), unexplained fever for ≥1 month (28%), recurrent or persistent diarrhea for ≥1 month (22%), and oral candidiasis (20%). Three women discovered their HIV status during an antenatal visit.

### History of HIV testing

Apart from the test that led to HIV diagnosis, 74 (11%) patients had been tested for HIV during their lifetime and 57 (8%) within the last 3 years. Among the latter, the median number of HIV tests performed within the previous 3 years was 1 (IQR:1–2). Their median CD4 count at HIV diagnosis was higher than in patients with no or a > 3-year history of HIV testing (336 versus 156 cells/mm^3^, *p* <  0.0001).

### Contacts with the healthcare system during the 3 years prior to HIV diagnosis

Over this time period, 61% of patients consulted at least once a general practitioner (GP), 32% a specialist physician, 31% a pharmacist, 12% an emergency medical service and 12% were admitted to hospital. As a result, 187 (29%) patients had no contact with the healthcare system over this 3-year period. Men were more likely not to seek medical care than women (35% versus 24%, *p* = 0.002). Among the 463 patients who had at least one contact with the healthcare system, 81% visited a GP at least once a year.

### Missed opportunities for HIV testing in patients with behavioral indicators

The analysis of opportunities for testing in patients with behavioral indicators was carried out in the 256 patients who used care during the 3 years prior to HIV diagnosis and had at least one behavioral indicator. Missed opportunities for HIV testing according to behavioral indicators are shown in Table 2. As an example, of the 35 men who reported being MSM as a behavioral indicator, 83% did not address their sexual behavior with the healthcare provider, 6% addressed this with the care provider and were offered a test, while 11% were not offered a test even though they mentioned they had sex with men.

**Table 2:** Opportunities for HIV testing in patients who sought care for clinical indicators or with behavioural indicators in the three years prior to HIV diagnosis

	Patients concerned n	Indicator reported to physician n (%)	HIV test proposal at the first visit n (%)
**Behavioural indicator (n = 463)**			
Multiple sex partners	185	21 (11%)	10 (48%)
Condomless sex with casual partners	166	25 (15%)	18 (72%)
Men who have sex with men	35	6 (17%)	2 (33%)
**Clinical indicator (n = 323)**			
Unexplained weight loss ≥10%	184	94 (51%)	4 (4%)
Unexplained fever for ≥1 month	112	83 (74%)	5 (6%)
Oral candidiasis	109	42 (39%)	4 (10%)
Sexually transmitted infections	96	72 (75%)	8 (11%)
Oral herpes	80	27 (34%)	0
Seborrheic dermatitis	57	21 (37%)	0
Prurigo	45	25 (56%)	0
Generalized lymphadenopathy	41	27 (66%)	3 (11%)
Recurrent diarrhoea ≥1 month	34	30 (88%)	3 (10%)
Recurrent bacterial infections	31	28 (90%)	2 (7%)
Varicella zoster	31	29 (94%)	1 (3%)
Community-acquired pneumonia	30	27 (90%)	0
Pulmonary tuberculosis	25	23 (92%)	3 (13%)
Onychomycosis	20	5 (25%)	0

### Missed opportunities for HIV testing in patients with clinical indicators

Almost 900 HIV-related clinical conditions were reported during the period of probable infection, corresponding to 323 patients who used care and had ≥1 clinical indicator during the previous 3 years (median number of clinical indicators per person: 2, IQR:1–4). The most frequent conditions reported were unexplained weight loss ≥10% (57%), unexplained fever for ≥1 month (35%), oral candidiasis (34%), and ≥ 1 sexually transmitted infection (30%). Testing opportunities for each clinical indicator are detailed in Table 2. Overall, among the 323 patients with ≥1 clinical indicator, 22% did not seek care for any of these indicator conditions, and 9% sought care and were offered a test at the first visit. The remaining 69% were not offered a test when they sought care. Considering only people who sought care, 88% had a missed opportunity for HIV testing.

The 29 patients who were offered an HIV test were compared to the 223 patients who were not when they sought care for clinical indicators (see Fig. S2). The only significant statistical association was that patients with a history of HIV testing in their lifetime were less likely to have a missed opportunity for HIV testing (at least one HIV test during lifetime: 7% among those who were not offered a test versus 24% among those who were, *p* = 0.009).

The 252 patients who sought care for HIV-related clinical indicators were compared to the 71 patients who did not (see Fig. S3). Living in an urban area rather than in the countryside was the only factor associated with access to care in the case of clinical indicators (rural place of residence: 82% among those who sought care versus 70% among those who did not, *p* = 0.03).

## Discussion

In Morocco, this multicenter study revealed that a high proportion of people newly diagnosed with HIV could have been tested earlier due to the presence of HIV-related high-risk behavioral and/or clinical conditions. First, nearly two out of three patients attended healthcare facilities during the period of probable HIV infection. Many of those with HIV indicators who sought care received no HIV test offer. For instance, 83% of MSM did not address their sexual behavior with the healthcare provider during this visit. Among the remaining MSM who had an opportunity for HIV testing, only one out of three was offered a test. In the same way, 69% of people who sought care for HIV-related clinical conditions were not offered an HIV test. Healthcare providers missed many opportunities to discover that their patients were living with HIV.

To our knowledge, this study is the first to describe the medical history of people infected with HIV before their HIV diagnosis in Morocco. In identifying all contacts with care, there was no computerized medical file common to the different health facilities. Therefore, medical information had to be collected retrospectively from the patients themselves, so the study may have suffered from recall bias leading to underestimation of the number of missed opportunities. To limit this bias, patients diagnosed with HIV more than 6 months before were excluded and the data collection period was restricted to 3 years prior to HIV diagnosis. Participants were asked about all clinical conditions of interest in our study, and a list of care facilities was drawn up to help them remember.

By directly interviewing patients, we investigated the overall patient trajectory through the healthcare system. Unlike some studies conducted in large urban hospitals in the United States and based on medical chart reviews [13, 16], we were able to detect HIV indicators whatever the access to care or the healthcare facility. This is all the more essential in a country where people often have access to care outside the hospital, mainly through private doctors or pharmacists.

Our study was based on a physician-administered questionnaire to help patients retrace their medical history, which consequently may have been subject to a social desirability bias concerning at-risk behaviors and sensitive issues that might be under-reported. Only 8% of participants (*n* = 51) reported being MSM, whereas it was estimated that nearly 15% of new HIV infections in Morocco occurred among MSM in 2010 [17]. Homosexuality was stigmatized in Morocco at this time and this may have had a negative impact on global health, HIV prevention, and retention in HIV care [18, 19]. Moreover, the National Strategic Plan against AIDS 2012–2016 [13] aimed to reduce discrimination and stigma, especially against MSM within healthcare settings [20].

Comparing our results with those of other studies was quite difficult because assessment of missed opportunities differed according to time-windows for retrospective detection of HIV testing opportunities, study designs and the populations considered [6–9, 21, 22]. One study in France in 2010 [6] and one in Ivory Coast in 2014 [7] revealed 82 and 77% of missed opportunities for HIV testing in people who sought care for an HIV-related clinical condition, respectively; this proportion is even higher in Morocco (88%).

These people with a missed opportunity had low CD4 counts at diagnosis (median: 97 cells/mm^3^, see Table S1) suggesting they were probably already infected with HIV at the time of the contact. As in other studies on the topic [6, 9, 23], whatever the clinical indicator, the proportion of HIV testing offered was very low. All people who sought care for herpes zoster or community-acquired pneumonia had a missed opportunity for HIV testing. Physicians’ knowledge of HIV clinical indicators should be strengthened.

Behavioral indicators were rarely addressed with the healthcare provider. On the one hand, patients may not have mentioned their behavior – multiple sex partners, condomless sex with casual partners or MSM – for fear of stigma or discrimination, or because they did not consider themselves to be at risk [24]. On the other hand, the physician did not ask about at-risk behaviors and offer a test. Studies that assessed the barriers to physicians offering HIV testing have highlighted difficulties in addressing sexuality with patients [25–28]. Our study showed that people who had been tested once in their lifetime were less likely to have a missed opportunity for testing compared to those who had never been tested before. This may be explained by the counseling associated with testing, which provided a better understanding of HIV transmission routes, and perception of risk-taking. Another key population very affected by HIV in Morocco is FSWs. No information was collected on sex work, but some women, who represented 11% of the study population, had a median of 40 partners (IQR: 20–100) and also reported more sexually transmitted infections and more behavioral indicators than others, but as many missed opportunities as others (data not shown). They were also more likely to have been tested for HIV during their lifetime.

In 2012, the Moroccan Ministry of Health implemented a new policy which aimed to strengthen HIV counseling and testing through a higher number of voluntary testing centers and the implementation of a mobile testing strategy [29]. Access to HIV testing has increased significantly with the introduction of community-based testing through rapid tests [30]. Of the estimated 20,000 people living with HIV in 2017, 70% were aware of their HIV status (versus 22% in 2010) [31]. This good result illustrates the need for sustained community-based HIV testing by lay providers to reach MSM and FSWs, who are still the populations most vulnerable to HIV infection [32, 33], and people in rural areas. To further strengthen and extend HIV testing services and overcome barriers to HIV testing, testing could be proposed in pharmacies to reach a large population given that pharmacists were consulted by 31% of our study population. The future implementation of self-testing in Morocco could also help to reach high-risk population groups, especially in a context of homosexuality and HIV stigmatization [34]. In addition, to curb the epidemic, the HIV testing strategies should be completed with strategies like treatment as prevention (TasP) or pre-exposure prophylaxis (PrEP) to prevent onwards transmission.

## Conclusions

In Morocco, among people who were unaware of their HIV infection and sought care, many opportunities for HIV testing, based on at-risk behaviors or clinical indicators, were missed. This highlights the need to improve the recognition of HIV clinical indicators by physicians, further expand community-based HIV testing by lay providers, and implement self-testing to increase accessibility and privacy.

## Supplementary Information

**Additional file 1: Fig. S1.** Distribution of HIV/AIDS cases in Morocco by region, 2005–2009.

**Additional file 2: Fig. S2.** Characteristics of patients who sought care for a clinical indicator in the three years prior to HIV diagnosis according to whether or not (missed opportunity) the care provider offered them an HIV test (*n* = 252).

**Additional file 3: Fig. S3.** Characteristics of patients reporting at least one clinical indicator in the three years prior to HIV diagnosis according to whether or not they sought care for this indicator (*n* = 323).

## Data Availability

All relevant data are within the manuscript and its Supporting Information file. The questionnaire and all data are available on request from the corresponding author.

## Abbreviations

AIDS: Acquired immunodeficiency syndrome
CD4: Lymphocyte T CD4
FSW: Female sex worker
GP: General practitioner
HIV: Human immunodeficiency virus
IQR: Interquartile range
MSM: Man who have sex with man
PrEP: Pre-exposure prophylaxis
PWID: People who inject drugs
TasP: Treatment as prevention

## References

[1] Palella FJ, Delaney KM, Moorman AC, Loveless MO, Fuhrer J, Satten GA (1998). Declining morbidity and mortality among patients with advanced human immunodeficiency virus infection. HIV outpatient study investigators. N Engl J Med.

[2] Cohen MS, Chen YQ, McCauley M, Gamble T, Hosseinipour MC, Kumarasamy N (2011). Prevention of HIV-1 infection with early antiretroviral therapy. N Engl J Med.

[3] Admou B, Elharti E, Oumzil H, Addebbous A, Amine M, Zahlane K (2010). Clinical and immunological status of a newly diagnosed HIV positive population, in Marrakech, Morocco. Afr Health Sci.

[4] OMS, ONUSIDA (2007). Guide du conseil et du dépistage du VIH à l’initiative du soignant dans les établissements de santé.

[5] Mumtaz G, Hilmi N, Zidouh A, El Rhilani H, Alami K, Bennani A (2010). HIV modes of transmission analysis in Morocco.

[6] Champenois K, Cousien A, Cuzin L, Le Vu S, Deuffic-Burban S, Lanoy E (2013). Missed opportunities for HIV testing in newly-HIV-diagnosed patients, a cross sectional study. BMC Infect Dis.

[7] Inghels M, Niangoran S, Minga A, Yoboue JM, Dohoun L, Yao A (2017). Missed opportunities for HIV testing among newly diagnosed HIV-infected adults in Abidjan, Côte d’Ivoire. PLOS ONE.

[8] Agustí C, Montoliu A, Mascort J, Carrillo R, Almeda J, Elorza JM (2016). Missed opportunities for HIV testing of patients diagnosed with an indicator condition in primary care in Catalonia. Spain Sex Transm Infect.

[9] Liddicoat RV, Horton NJ, Urban R, Maier E, Christiansen D, Samet JH (2004). Assessing missed opportunities for HIV testing in medical settings. J Gen Intern Med.

[10] Sullivan AK, Raben D, Reekie J, Rayment M, Mocroft A, Esser S (2013). Feasibility and effectiveness of Indicator condition-guided testing for HIV: results from HIDES I (HIV Indicator diseases across Europe study). PLoS One.

[11] Raben D, Mocroft A, Rayment M, Mitsura VM, Hadziosmanovic V, Sthoeger ZM (2015). Auditing HIV testing rates across Europe: results from the HIDES 2 study. PLoS One.

[12] Raben D, Sullivan AK, Mocroft A, Kutsyna G, Hadžiosmanović V, Vassilenko A (2019). Improving the evidence for indicator condition guided HIV testing in Europe: Results from the HIDES II Study – 2012 – 2015. PLoS One.

[13] du Maroc R, de la Santé M (2012). Plan Stratégique National de Lutte contre le SIDA 2012–2016.

[14] Abu-Raddad LJ, Akala FA, Semini I, Riedner G, Wilson D, Tawil O (2010). Characterizing the HIV/AIDS epidemic in the Middle East and North Africa : time for strategic action.

[15] WHO (2006). WHO case definitions of HIV for surveillance and revised clinical staging and immunological classification of HIV-related disease in adults and children.

[16] Freedberg KA, Samet JH (1999). Think HIV: Why physicians should lower their threshold for HIV testing. Arch Intern Med.

[17] HIV Indicator Conditions (2012). Guidance for implementing HIV testing in Adults in Health Care Settings.

[18] Klein D, Hurley LB, Merrill D, Quesenberry CP (2003). Consortium for HIV/AIDS interregional research. Review of medical encounters in the 5 years before a diagnosis of HIV-1 infection: implications for early detection. J Acquir Immune Defic Syndr.

[19] Katz IT, Ryu AE, Onuegbu AG, Psaros C, Weiser SD, Bangsberg DR (2013). Impact of HIV-related stigma on treatment adherence: systematic review and meta-synthesis. J Int AIDS Soc.

[20] Mumtaz GR, Kouyoumjian SP, Hilmi N, Zidouh A, Rhilani HE, Alami K (2013). The distribution of new HIV infections by mode of exposure in Morocco. Sex Transm Infect.

[21] du Maroc R, de la Santé M (2016). Revue de l’environnement législatif et réglementaire lié au VIH/Sida au Maroc.

[22] UNAIDS. UNAIDS data 2018. 2018. https://www.unaids.org/en/resources/documents/2018/unaids-data-2018 (accessed 12 Dec 2020).

[23] Lhopitallier L, Moulin E, Hugli O, Cavassini M, Darling KEA (2018). Missed opportunities for HIV testing among patients newly presenting for HIV care at a Swiss university hospital: a retrospective analysis. BMJ Open.

[24] Espinel M, Belza MJ, Cabeza-de-Vaca C, Arranz B, Guerras JM, Garcia-Soltero J (2018). Indicator condition based HIV testing: missed opportunities for earlier diagnosis in men who have sex with men. Enfermedades Infecc Microbiol Clin Engl Ed.

[25] Ellis S, Curtis H, Ong EL (2012). HIV diagnoses and missed opportunities. Results of the British HIV Association (BHIVA) National Audit 2010. Clin Med.

[26] Deblonde J, De Koker P, Hamers FF, Fontaine J, Luchters S, Temmerman M (2010). Barriers to HIV testing in Europe: a systematic review. Eur J Pub Health.

[27] Epstein RM (1998). Awkward moments in patient-physician communication about HIV risk. Ann Intern Med.

[28] Burke RC, Sepkowitz KA, Bernstein KT, Karpati AM, Myers JE, Tsoi BW (2007). Why don’t physicians test for HIV? A review of the US literature. AIDS..

[29] du Maroc R, de la Santé M (2009). Plan Stratégique National de Lutte contre le SIDA 2007–2011. Ministère de la Santé.

[30] UNAIDS. Morocco. https://www.unaids.org/en/regionscountries/countries/morocco (accessed 12 Dec 2020).

[31] du Maroc R, de la Santé M (2017). Situation épidémiologique et progrès de la riposte nationale au Sida.

[32] Spire B, de Zoysa I, Himmich H (2008). HIV prevention: what have we learned from community experiences in concentrated epidemics?. J Int AIDS Soc.

[33] WHO (2015). Consolidated guidelines on HIV testing services.

[34] WHO (2016). Guidelines on HIV self-testing and partner notification.

