# Supplementary material for: Missed opportunities for HIV testing in patients newly diagnosed with HIV in Morocco
Source: BMC Infect Dis. 2021 Jan 11;21:48. doi: 10.1186/s12879-020-05711-2 (PMC7802172; doi:10.1186/s12879-020-05711-2)
Supplement: Supplementary file 1 — Additional file 1: Fig. S1. Distribution of HIV/AIDS cases in Morocco by region, 2005–2009. [file 12879_2020_5711_MOESM1_ESM.docx]

**Missed opportunities for HIV testing in patients newly diagnosed with HIV in Morocco**

**Supporting Information**

**Figure S1. Distribution of HIV/AIDS cases in Morocco by region, 2005-2009.**


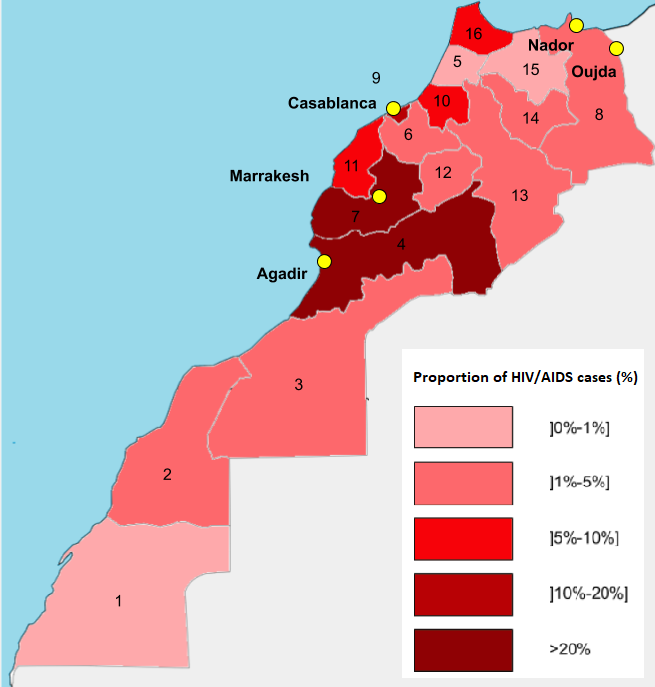


Regions: 1.Oued Ed-Dahab-Lagouira, 2.Laâyoune-Boujdour-Sakia el Hamra, 3.Guelmim-Es Smara, 4.Sous-Massa-Drâa, 5.Gharb-Chrarda-Beni Hssen, 6.Chaouia-Ouardigha, 7.Marrakech-Tensift-Al Haouz, 8.Oriental, 9.Grand Casablanca, 10.Rabat-Salé-Zemmour-Zaër, 11.Doukhala-Abda, 12.Tadla-Azilal, 13.Meknès-Tafilalet, 14.Fès-Boulemane, 15.Taza-Al Hoceima-Taounate, 16.Tanger-Tétouan.

Study centres: Agadir (1), Casablanca (1), Marrakesh (2), Nador (1), Oujda (1).

This figure has been made from a blank map (https://d-maps.com) with prevalence data from Mumtaz et al. 2010 (<http://www.unaids.org/en/media/unaids/contentassets/documents/countryreport/2010/201008_MOT_Morocco_en.pdf>).
