# Supplementary material for: Missed opportunities for HIV testing in patients newly diagnosed with HIV in Morocco
Source: BMC Infect Dis. 2021 Jan 11;21:48. doi: 10.1186/s12879-020-05711-2 (PMC7802172; doi:10.1186/s12879-020-05711-2)
Supplement: Supplementary file 2 — Additional file 2: Fig. S2. Characteristics of patients who sought care for a clinical indicator in the three years prior to HIV diagnosis according to whether or not (missed opportunity) the care provider offered them an HIV test (n = 252). [file 12879_2020_5711_MOESM2_ESM.docx]

**Missed opportunities for HIV testing in patients newly diagnosed with HIV in Morocco**

**Supporting Information**

**Figure S2. Characteristics of patients who sought care for a clinical indicator in the three years prior to HIV diagnosis according to whether or not (missed opportunity) the care provider offered them an HIV test (n=252).**


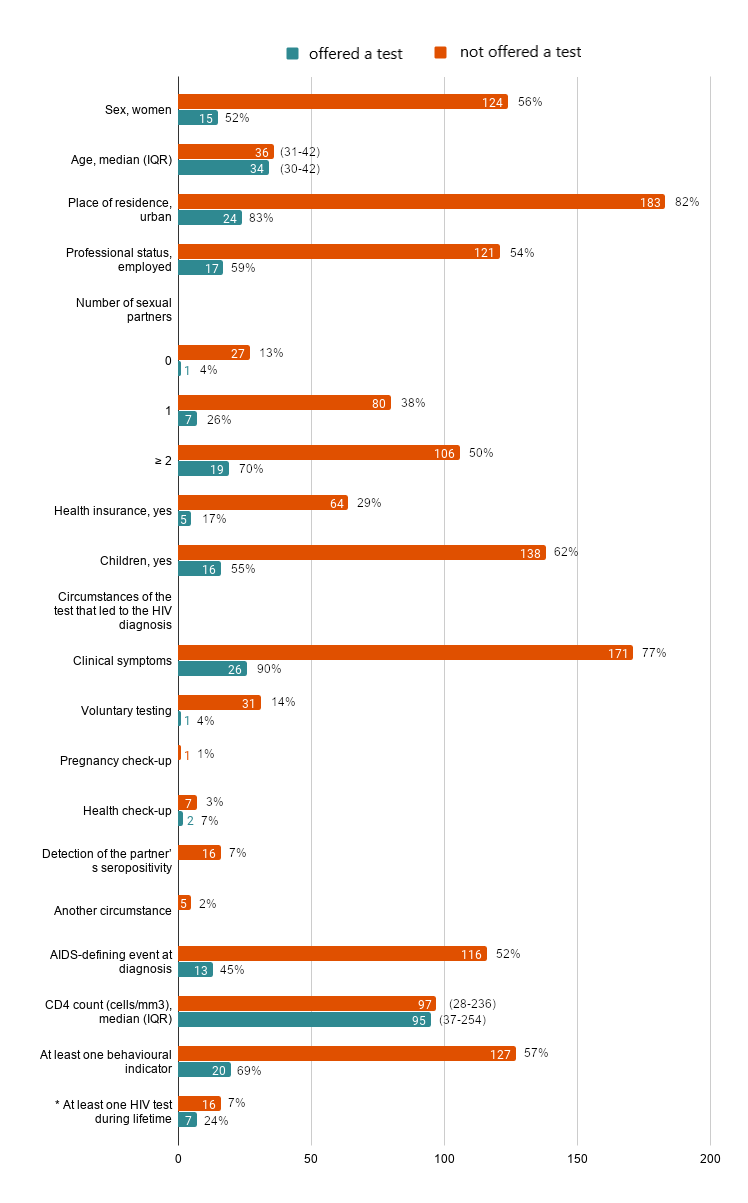


IQR: interquartile range.

* Characteristics of the two groups of patients were compared using Chi2 tests. Only one comparison was significant. At least one HIV test during lifetime: 7% among those who were not offered a test versus 24% among those who were, p=0.009.
