# Supplementary material for: Missed opportunities for HIV testing in patients newly diagnosed with HIV in Morocco
Source: BMC Infect Dis. 2021 Jan 11;21:48. doi: 10.1186/s12879-020-05711-2 (PMC7802172; doi:10.1186/s12879-020-05711-2)
Supplement: Supplementary file 3 — Additional file 3: Fig. S3. Characteristics of patients reporting at least one clinical indicator in the three years prior to HIV diagnosis according to whether or not they sought care for this indicator (n = 323). [file 12879_2020_5711_MOESM3_ESM.docx]

**Missed opportunities for HIV testing in patients newly diagnosed with HIV in Morocco**

**Supporting Information**

**Figure S3. Characteristics of patients reporting at least one clinical indicator in the three years prior to HIV diagnosis according to whether or not they sought care for this indicator (n=323).**

**
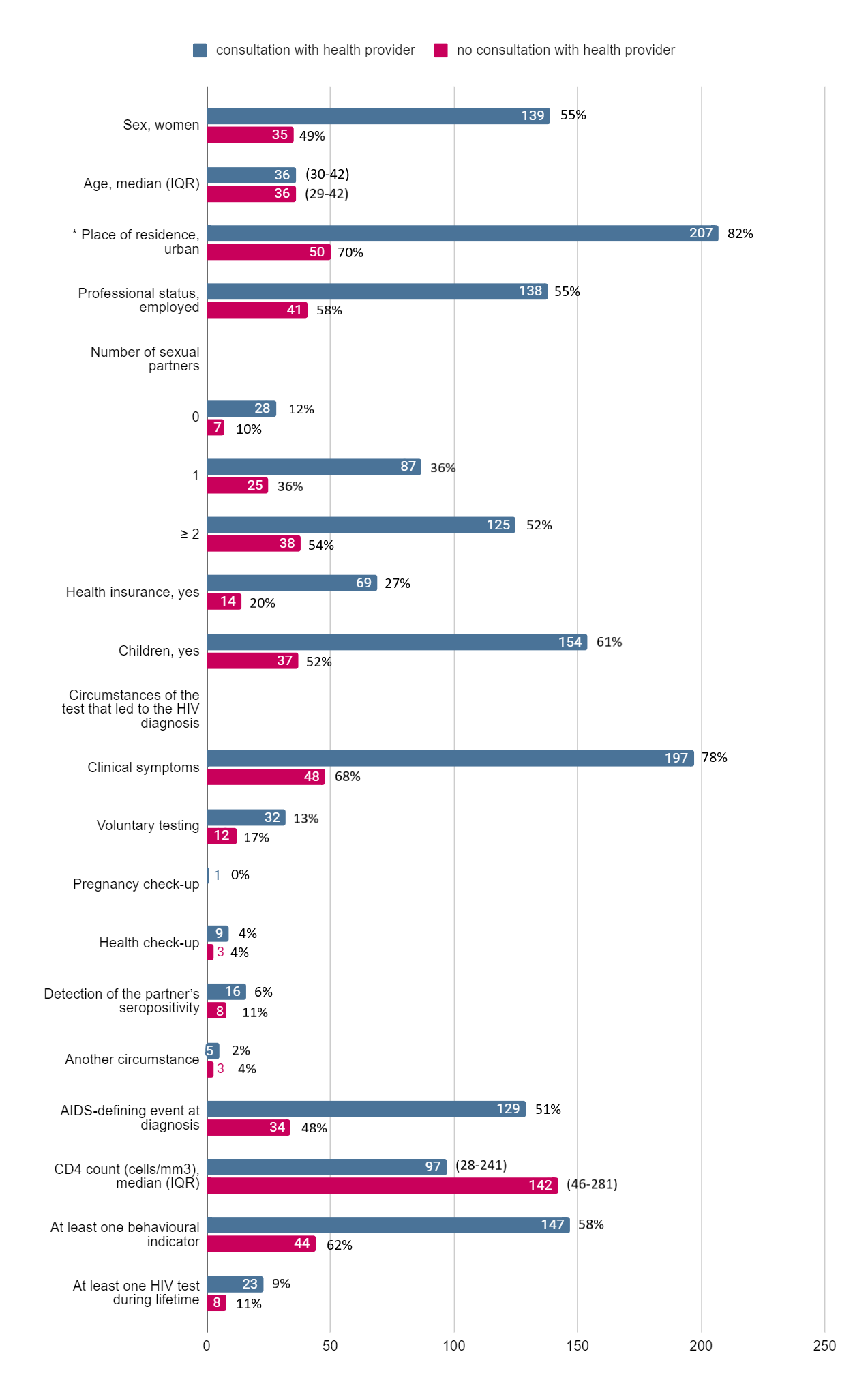
**

IQR: interquartile range.

* Characteristics of the two groups of patients were compared using Chi2 tests. Only one comparison was significant. Rural place of residence: 82% among those who sought care versus 70% among those who did not, p=0.03.
